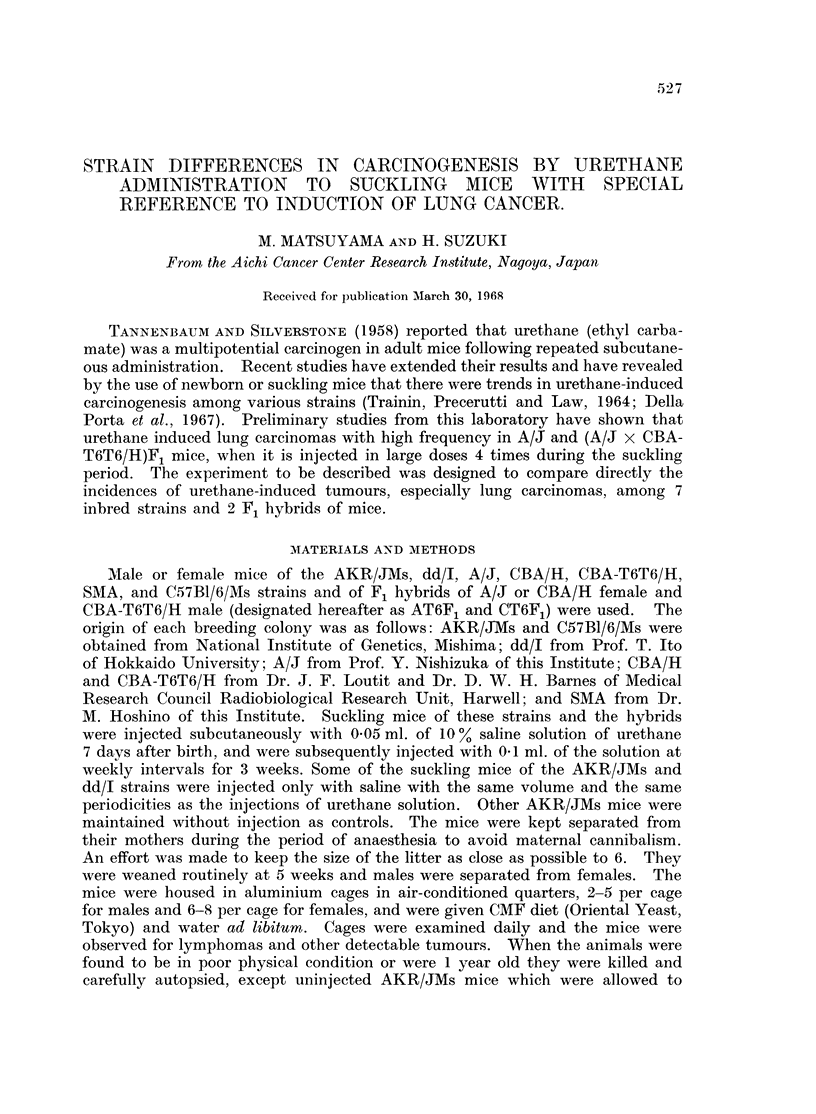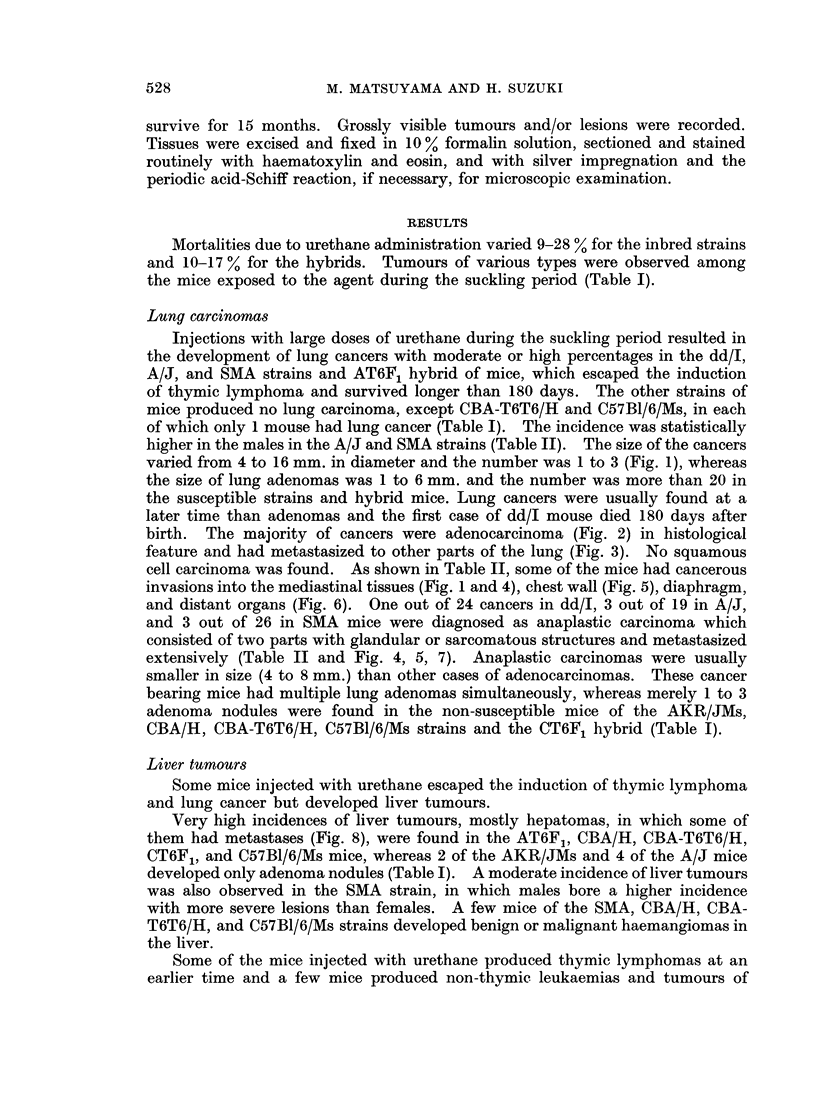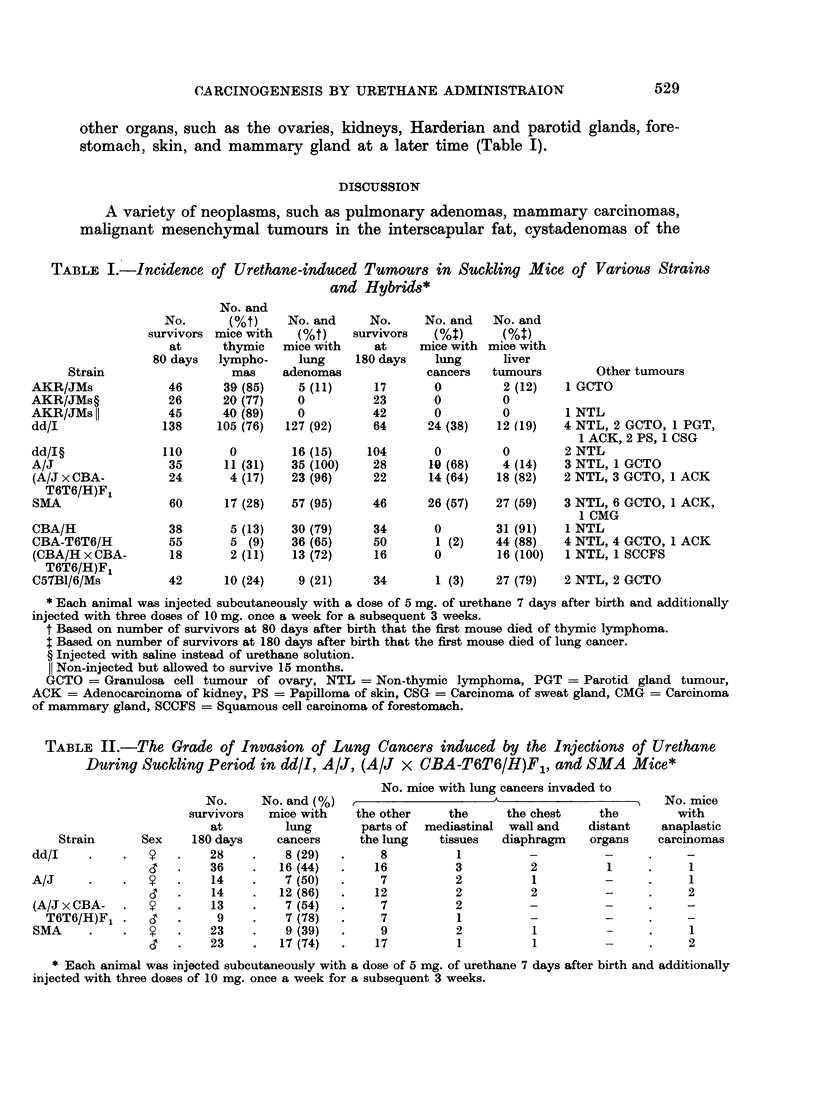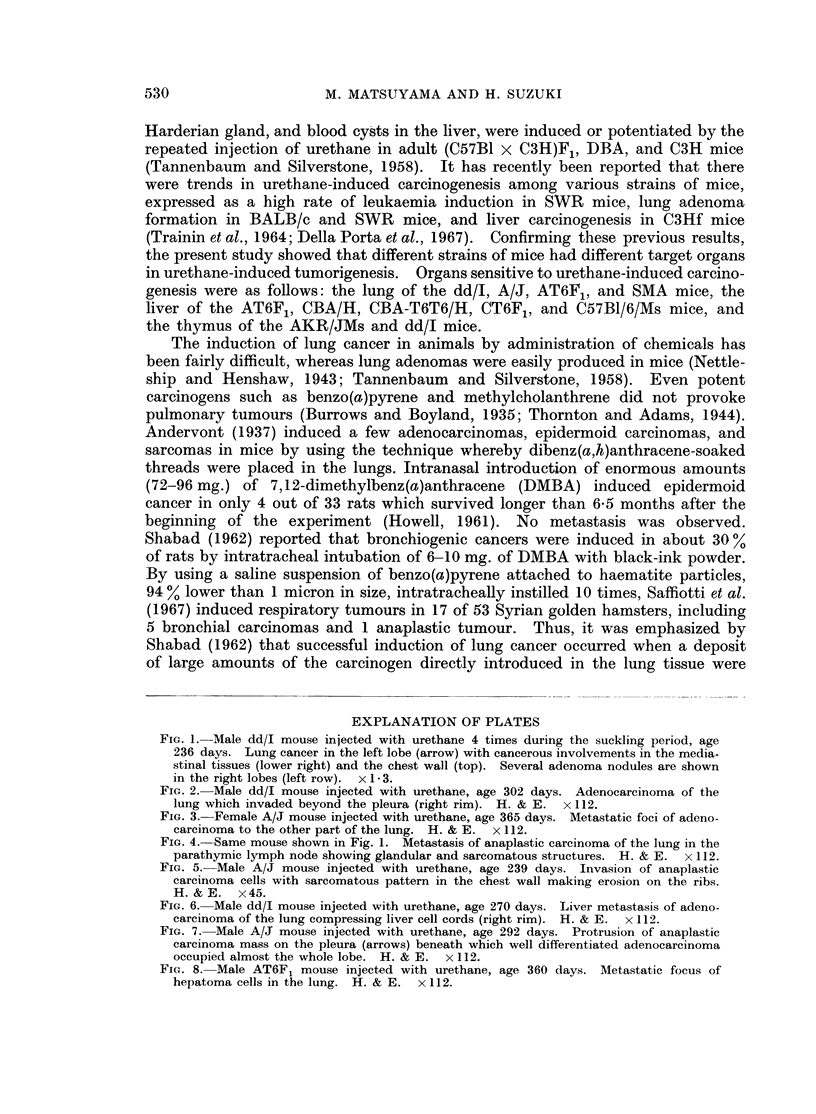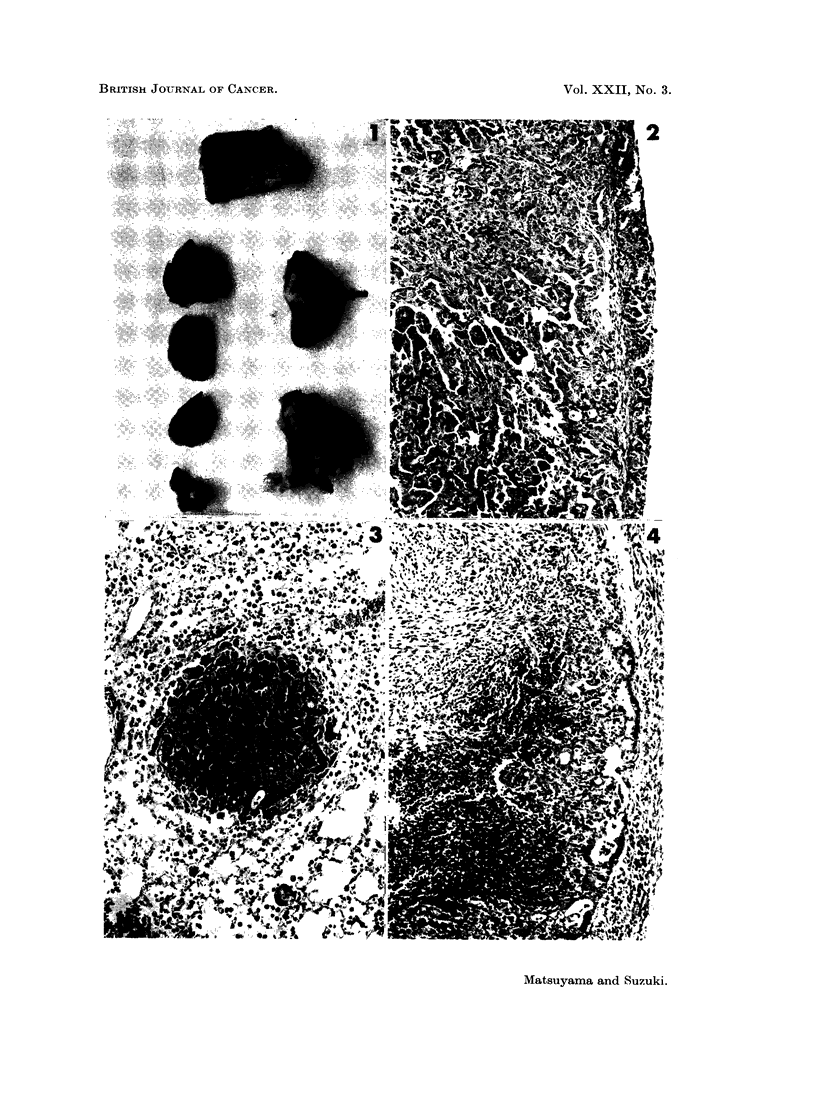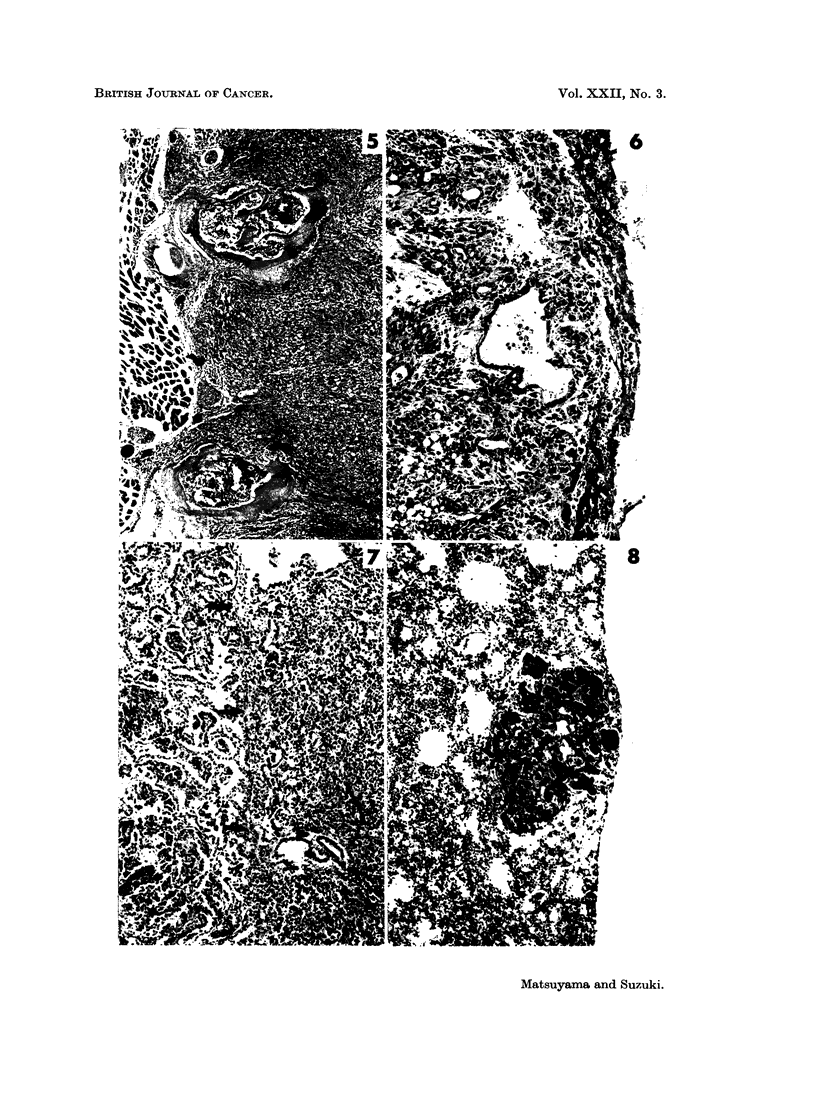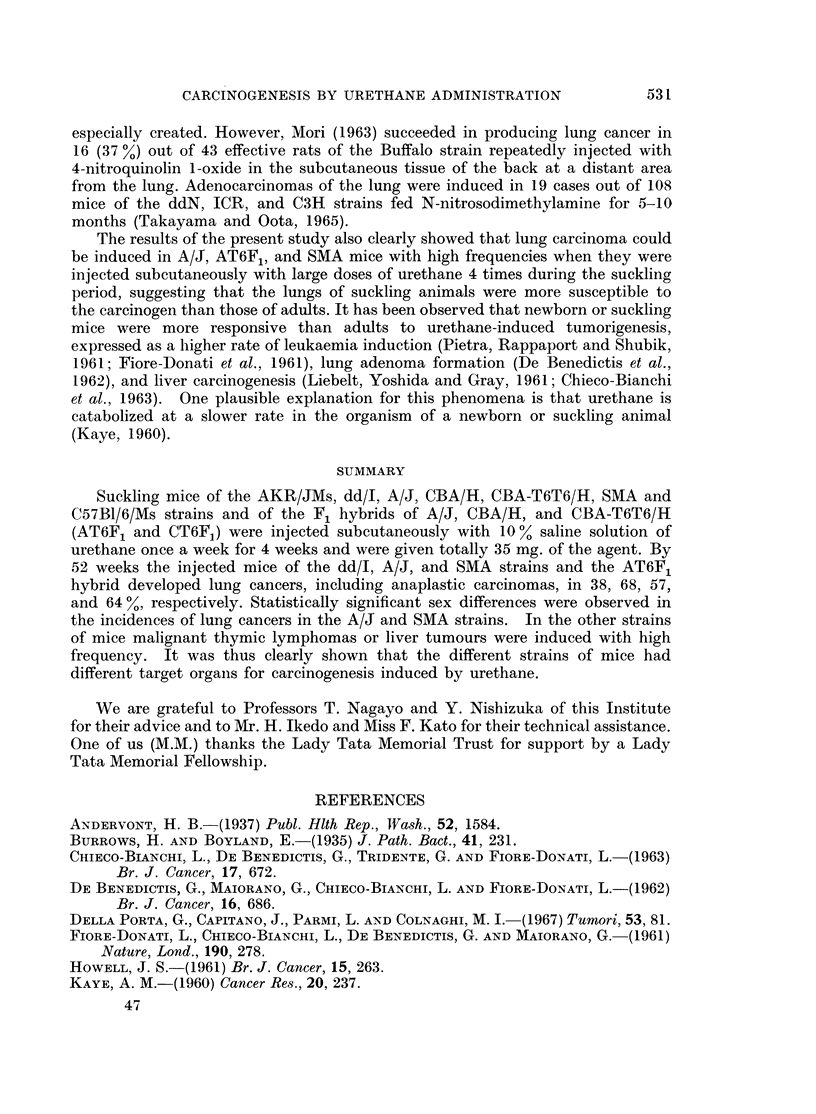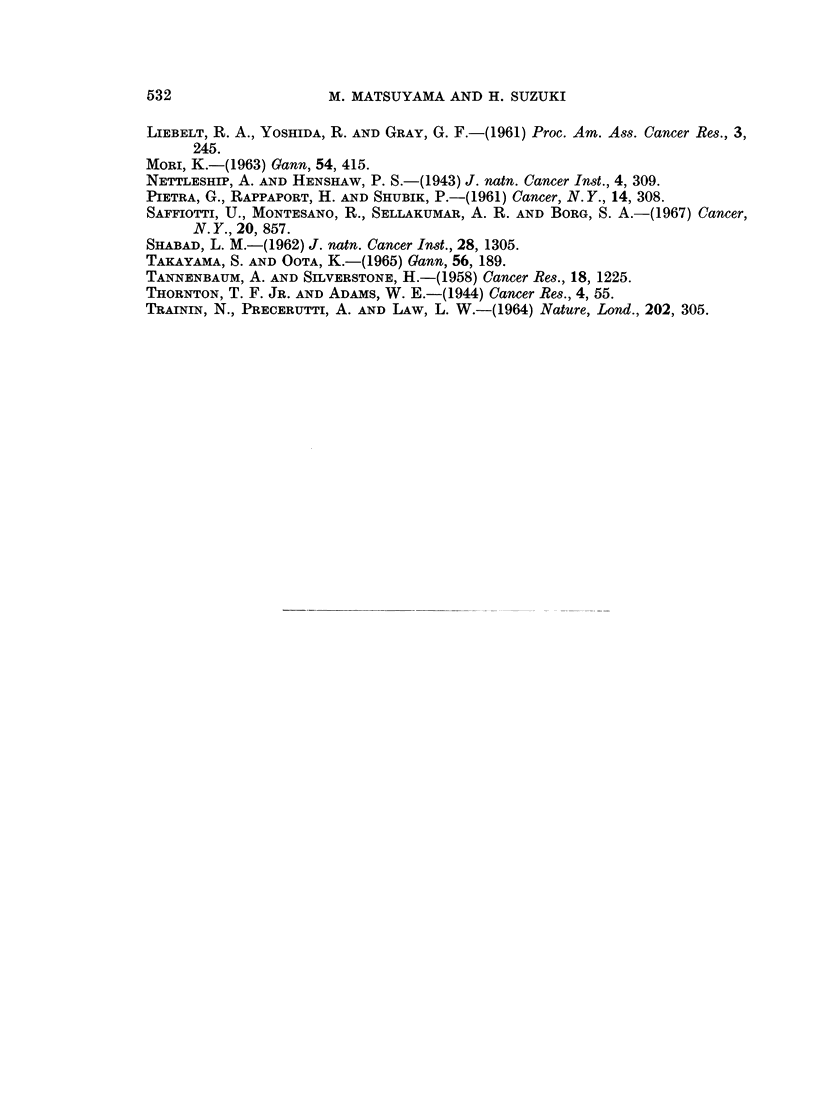# Strain differences in carcinogenesis by urethane administration to suckling mice with special reference to induction of lung cancer.

**DOI:** 10.1038/bjc.1968.63

**Published:** 1968-09

**Authors:** M. Matsuyama, H. Suzuki

## Abstract

**Images:**


### STRAIN DIFFERENCES IN CARCINOGENESIS BY URETHANE ADMINISTRATION TO SUCKLING MICE WITH SPECIAL REFERENCE TO INDUCTION OF LUNG CANCER.

M. MATSUYAMA AND H. SUZUKI

*From the Aichi Cancer Center Research Institute, Nagoya, Japan*

Received for publication March 30, 1968

TANNENBAUM AND SILVERSTONE (1958) reported that urethane (ethyl carbamate) was a multipotential carcinogen in adult mice following repeated subcutaneous administration. Recent studies have extended their results and have revealed by the use of newborn or suckling mice that there were trends in urethane-induced carcinogenesis among various strains (Trainin, Precerutti and Law, 1964; Della Porta *et al.*, 1967). Preliminary studies from this laboratory have shown that urethane induced lung carcinomas with high frequency in A/J and (A/J × CBA-T6T6/H)$F_1$ mice, when it is injected in large doses 4 times during the suckling period. The experiment to be described was designed to compare directly the incidences of urethane-induced tumours, especially lung carcinomas, among 7 inbred strains and 2 $F_1$ hybrids of mice.

### MATERIALS AND METHODS

Male or female mice of the AKR/JMs, dd/I, A/J, CBA/H, CBA-T6T6/H, SMA, and C57Bl/6/Ms strains and of $F_1$ hybrids of A/J or CBA/H female and CBA-T6T6/H male (designated hereafter as AT6$F_1$ and CT6$F_1$) were used. The origin of each breeding colony was as follows: AKR/JMs and C57Bl/6/Ms were obtained from National Institute of Genetics, Mishima; dd/I from Prof. T. Ito of Hokkaido University; A/J from Prof. Y. Nishizuka of this Institute; CBA/H and CBA-T6T6/H from Dr. J. F. Loutit and Dr. D. W. H. Barnes of Medical Research Council Radiobiological Research Unit, Harwell; and SMA from Dr. M. Hoshino of this Institute. Suckling mice of these strains and the hybrids were injected subcutaneously with 0·05 ml. of 10% saline solution of urethane 7 days after birth, and were subsequently injected with 0·1 ml. of the solution at weekly intervals for 3 weeks. Some of the suckling mice of the AKR/JMs and dd/I strains were injected only with saline with the same volume and the same periodicities as the injections of urethane solution. Other AKR/JMs mice were maintained without injection as controls. The mice were kept separated from their mothers during the period of anaesthesia to avoid maternal cannibalism. An effort was made to keep the size of the litter as close as possible to 6. They were weaned routinely at 5 weeks and males were separated from females. The mice were housed in aluminium cages in air-conditioned quarters, 2–5 per cage for males and 6–8 per cage for females, and were given CMF diet (Oriental Yeast, Tokyo) and water *ad libitum.* Cages were examined daily and the mice were observed for lymphomas and other detectable tumours. When the animals were found to be in poor physical condition or were 1 year old they were killed and carefully autopsied, except uninjected AKR/JMs mice which were allowed to survive for 15 months. Grossly visible tumours and/or lesions were recorded. Tissues were excised and fixed in 10% formalin solution, sectioned and stained routinely with haematoxylin and eosin, and with silver impregnation and the periodic acid-Schiff reaction, if necessary, for microscopic examination.

### RESULTS

Mortalities due to urethane administration varied 9–28% for the inbred strains and 10–17% for the hybrids. Tumours of various types were observed among the mice exposed to the agent during the suckling period (Table I).

*Lung carcinomas*

Injections with large doses of urethane during the suckling period resulted in the development of lung cancers with moderate or high percentages in the dd/I, A/J, and SMA strains and $AT6F_1$ hybrid of mice, which escaped the induction of thymic lymphoma and survived longer than 180 days. The other strains of mice produced no lung carcinoma, except CBA-T6T6/H and C57Bl/6/Ms, in each of which only 1 mouse had lung cancer (Table I). The incidence was statistically higher in the males in the A/J and SMA strains (Table II). The size of the cancers varied from 4 to 16 mm. in diameter and the number was 1 to 3 (Fig. 1), whereas the size of lung adenomas was 1 to 6 mm. and the number was more than 20 in the susceptible strains and hybrid mice. Lung cancers were usually found at a later time than adenomas and the first case of dd/I mouse died 180 days after birth. The majority of cancers were adenocarcinoma (Fig. 2) in histological feature and had metastasized to other parts of the lung (Fig. 3). No squamous cell carcinoma was found. As shown in Table II, some of the mice had cancerous invasions into the mediastinal tissues (Fig. 1 and 4), chest wall (Fig. 5), diaphragm, and distant organs (Fig. 6). One out of 24 cancers in dd/I, 3 out of 19 in A/J, and 3 out of 26 in SMA mice were diagnosed as anaplastic carcinoma which consisted of two parts with glandular or sarcomatous structures and metastasized extensively (Table II and Fig. 4, 5, 7). Anaplastic carcinomas were usually smaller in size (4 to 8 mm.) than other cases of adenocarcinomas. These cancer bearing mice had multiple lung adenomas simultaneously, whereas merely 1 to 3 adenoma nodules were found in the non-susceptible mice of the AKR/JMs, CBA/H, CBA-T6T6/H, C57Bl/6/Ms strains and the $CT6F_1$ hybrid (Table I).

*Liver tumours*

Some mice injected with urethane escaped the induction of thymic lymphoma and lung cancer but developed liver tumours.

Very high incidences of liver tumours, mostly hepatomas, in which some of them had metastases (Fig. 8), were found in the $AT6F_1$, CBA/H, CBA-T6T6/H, $CT6F_1$, and C57Bl/6/Ms mice, whereas 2 of the AKR/JMs and 4 of the A/J mice developed only adenoma nodules (Table I). A moderate incidence of liver tumours was also observed in the SMA strain, in which males bore a higher incidence with more severe lesions than females. A few mice of the SMA, CBA/H, CBA-T6T6/H, and C57Bl/6/Ms strains developed benign or malignant haemangiomas in the liver.

Some of the mice injected with urethane produced thymic lymphomas at an earlier time and a few mice produced non-thymic leukaemias and tumours of other organs, such as the ovaries, kidneys, Harderian and parotid glands, forestomach, skin, and mammary gland at a later time (Table I).

### DISCUSSION

A variety of neoplasms, such as pulmonary adenomas, mammary carcinomas, malignant mesenchymal tumours in the interscapular fat, cystadenomas of the

TABLE I.—*Incidence of Urethane-induced Tumours in Suckling Mice of Various Strains and Hybrids**

| Strain | No. survivors at 80 days | No. and (%†) mice with thymic lymphomas | No. and (%†) mice with lung adenomas | No. survivors at 180 days | No. and (%‡) mice with lung cancers | No. and (%‡) mice with liver tumours | Other tumours |
|---|---|---|---|---|---|---|---|
| AKR/JMs | 46 | 39 (85) | 5 (11) | 17 | 0 | 2 (12) | 1 GCTO |
| AKR/JMs§ | 26 | 20 (77) | 0 | 23 | 0 | 0 | |
| AKR/JMs‖ | 45 | 40 (89) | 0 | 42 | 0 | 0 | 1 NTL |
| dd/I | 138 | 105 (76) | 127 (92) | 64 | 24 (38) | 12 (19) | 4 NTL, 2 GCTO, 1 PGT, 1 ACK, 2 PS, 1 CSG |
| dd/I§ | 110 | 0 | 16 (15) | 104 | 0 | 0 | 2 NTL |
| A/J | 35 | 11 (31) | 35 (100) | 28 | 19 (68) | 4 (14) | 3 NTL, 1 GCTO |
| (A/J × CBA-T6T6/H)$F_1$ | 24 | 4 (17) | 23 (96) | 22 | 14 (64) | 18 (82) | 2 NTL, 3 GCTO, 1 ACK |
| SMA | 60 | 17 (28) | 57 (95) | 46 | 26 (57) | 27 (59) | 3 NTL, 6 GCTO, 1 ACK, 1 CMG |
| CBA/H | 38 | 5 (13) | 30 (79) | 34 | 0 | 31 (91) | 1 NTL |
| CBA-T6T6/H | 55 | 5 (9) | 36 (65) | 50 | 1 (2) | 44 (88) | 4 NTL, 4 GCTO, 1 ACK |
| (CBA/H × CBA-T6T6/H)$F_1$ | 18 | 2 (11) | 13 (72) | 16 | 0 | 16 (100) | 1 NTL, 1 SCCFS |
| C57Bl/6/Ms | 42 | 10 (24) | 9 (21) | 34 | 1 (3) | 27 (79) | 2 NTL, 2 GCTO |

* Each animal was injected subcutaneously with a dose of 5 mg. of urethane 7 days after birth and additionally injected with three doses of 10 mg. once a week for a subsequent 3 weeks.
† Based on number of survivors at 80 days after birth that the first mouse died of thymic lymphoma.
‡ Based on number of survivors at 180 days after birth that the first mouse died of lung cancer.
§ Injected with saline instead of urethane solution.
‖ Non-injected but allowed to survive 15 months.
GCTO = Granulosa cell tumour of ovary, NTL = Non-thymic lymphoma, PGT = Parotid gland tumour, ACK = Adenocarcinoma of kidney, PS = Papilloma of skin, CSG = Carcinoma of sweat gland, CMG = Carcinoma of mammary gland, SCCFS = Squamous cell carcinoma of forestomach.

TABLE II.—*The Grade of Invasion of Lung Cancers induced by the Injections of Urethane During Suckling Period in dd/I, A/J, (A/J × CBA-T6T6/H)$F_1$, and SMA Mice**

| Strain | Sex | No. survivors at 180 days | No. and (%) mice with lung cancers | No. mice with lung cancers invaded to: the other parts of the lung | the mediastinal tissues | the chest wall and diaphragm | the distant organs | No. mice with anaplastic carcinomas |
|---|---|---|---|---|---|---|---|---|
| dd/I | ♀ | 28 | 8 (29) | 8 | 1 | – | – | – |
| | ♂ | 36 | 16 (44) | 16 | 3 | 2 | 1 | 1 |
| A/J | ♀ | 14 | 7 (50) | 7 | 2 | 1 | – | 1 |
| | ♂ | 14 | 12 (86) | 12 | 2 | 2 | – | 2 |
| (A/J × CBA-T6T6/H)$F_1$ | ♀ | 13 | 7 (54) | 7 | 2 | – | – | – |
| | ♂ | 9 | 7 (78) | 7 | 1 | – | – | – |
| SMA | ♀ | 23 | 9 (39) | 9 | 2 | 1 | – | 1 |
| | ♂ | 23 | 17 (74) | 17 | 1 | 1 | – | 2 |

* Each animal was injected subcutaneously with a dose of 5 mg. of urethane 7 days after birth and additionally injected with three doses of 10 mg. once a week for a subsequent 3 weeks.

Harderian gland, and blood cysts in the liver, were induced or potentiated by the repeated injection of urethane in adult (C57Bl × C3H)$F_1$, DBA, and C3H mice (Tannenbaum and Silverstone, 1958). It has recently been reported that there were trends in urethane-induced carcinogenesis among various strains of mice, expressed as a high rate of leukaemia induction in SWR mice, lung adenoma formation in BALB/c and SWR mice, and liver carcinogenesis in C3Hf mice (Trainin *et al.*, 1964; Della Porta *et al.*, 1967). Confirming these previous results, the present study showed that different strains of mice had different target organs in urethane-induced tumorigenesis. Organs sensitive to urethane-induced carcinogenesis were as follows: the lung of the dd/I, A/J, AT6$F_1$, and SMA mice, the liver of the AT6$F_1$, CBA/H, CBA-T6T6/H, CT6$F_1$, and C57Bl/6/Ms mice, and the thymus of the AKR/JMs and dd/I mice.

The induction of lung cancer in animals by administration of chemicals has been fairly difficult, whereas lung adenomas were easily produced in mice (Nettleship and Henshaw, 1943; Tannenbaum and Silverstone, 1958). Even potent carcinogens such as benzo(*a*)pyrene and methylcholanthrene did not provoke pulmonary tumours (Burrows and Boyland, 1935; Thornton and Adams, 1944). Andervont (1937) induced a few adenocarcinomas, epidermoid carcinomas, and sarcomas in mice by using the technique whereby dibenz(*a,h*)anthracene-soaked threads were placed in the lungs. Intranasal introduction of enormous amounts (72–96 mg.) of 7,12-dimethylbenz(*a*)anthracene (DMBA) induced epidermoid cancer in only 4 out of 33 rats which survived longer than 6·5 months after the beginning of the experiment (Howell, 1961). No metastasis was observed. Shabad (1962) reported that bronchiogenic cancers were induced in about 30% of rats by intratracheal intubation of 6–10 mg. of DMBA with black-ink powder. By using a saline suspension of benzo(*a*)pyrene attached to haematite particles, 94% lower than 1 micron in size, intratracheally instilled 10 times, Saffiotti *et al.* (1967) induced respiratory tumours in 17 of 53 Syrian golden hamsters, including 5 bronchial carcinomas and 1 anaplastic tumour. Thus, it was emphasized by Shabad (1962) that successful induction of lung cancer occurred when a deposit of large amounts of the carcinogen directly introduced in the lung tissue were

---

EXPLANATION OF PLATES

FIG. 1.—Male dd/I mouse injected with urethane 4 times during the suckling period, age 236 days. Lung cancer in the left lobe (arrow) with cancerous involvements in the mediastinal tissues (lower right) and the chest wall (top). Several adenoma nodules are shown in the right lobes (left row). ×1·3.

FIG. 2.—Male dd/I mouse injected with urethane, age 302 days. Adenocarcinoma of the lung which invaded beyond the pleura (right rim). H. & E. ×112.

FIG. 3.—Female A/J mouse injected with urethane, age 365 days. Metastatic foci of adenocarcinoma to the other part of the lung. H. & E. ×112.

FIG. 4.—Same mouse shown in Fig. 1. Metastasis of anaplastic carcinoma of the lung in the parathymic lymph node showing glandular and sarcomatous structures. H. & E. ×112.

FIG. 5.—Male A/J mouse injected with urethane, age 239 days. Invasion of anaplastic carcinoma cells with sarcomatous pattern in the chest wall making erosion on the ribs. H. & E. ×45.

FIG. 6.—Male dd/I mouse injected with urethane, age 270 days. Liver metastasis of adenocarcinoma of the lung compressing liver cell cords (right rim). H. & E. ×112.

FIG. 7.—Male A/J mouse injected with urethane, age 292 days. Protrusion of anaplastic carcinoma mass on the pleura (arrows) beneath which well differentiated adenocarcinoma occupied almost the whole lobe. H. & E. ×112.

FIG. 8.—Male AT6$F_1$ mouse injected with urethane, age 360 days. Metastatic focus of hepatoma cells in the lung. H. & E. ×112.

1
2
3
4

Matsuyama and Suzuki.

especially created. However, Mori (1963) succeeded in producing lung cancer in 16 (37 %) out of 43 effective rats of the Buffalo strain repeatedly injected with 4-nitroquinolin 1-oxide in the subcutaneous tissue of the back at a distant area from the lung. Adenocarcinomas of the lung were induced in 19 cases out of 108 mice of the ddN, ICR, and C3H strains fed N-nitrosodimethylamine for 5–10 months (Takayama and Oota, 1965).

The results of the present study also clearly showed that lung carcinoma could be induced in A/J, AT6$F_1$, and SMA mice with high frequencies when they were injected subcutaneously with large doses of urethane 4 times during the suckling period, suggesting that the lungs of suckling animals were more susceptible to the carcinogen than those of adults. It has been observed that newborn or suckling mice were more responsive than adults to urethane-induced tumorigenesis, expressed as a higher rate of leukaemia induction (Pietra, Rappaport and Shubik, 1961; Fiore-Donati *et al.*, 1961), lung adenoma formation (De Benedictis *et al.*, 1962), and liver carcinogenesis (Liebelt, Yoshida and Gray, 1961; Chieco-Bianchi *et al.*, 1963). One plausible explanation for this phenomena is that urethane is catabolized at a slower rate in the organism of a newborn or suckling animal (Kaye, 1960).

### SUMMARY

Suckling mice of the AKR/JMs, dd/I, A/J, CBA/H, CBA-T6T6/H, SMA and C57Bl/6/Ms strains and of the $F_1$ hybrids of A/J, CBA/H, and CBA-T6T6/H (AT6$F_1$ and CT6$F_1$) were injected subcutaneously with 10 % saline solution of urethane once a week for 4 weeks and were given totally 35 mg. of the agent. By 52 weeks the injected mice of the dd/I, A/J, and SMA strains and the AT6$F_1$ hybrid developed lung cancers, including anaplastic carcinomas, in 38, 68, 57, and 64 %, respectively. Statistically significant sex differences were observed in the incidences of lung cancers in the A/J and SMA strains. In the other strains of mice malignant thymic lymphomas or liver tumours were induced with high frequency. It was thus clearly shown that the different strains of mice had different target organs for carcinogenesis induced by urethane.

We are grateful to Professors T. Nagayo and Y. Nishizuka of this Institute for their advice and to Mr. H. Ikedo and Miss F. Kato for their technical assistance. One of us (M.M.) thanks the Lady Tata Memorial Trust for support by a Lady Tata Memorial Fellowship.

### REFERENCES

ANDERVONT, H. B.—(1937) *Publ. Hlth Rep., Wash.*, **52**, 1584.
BURROWS, H. AND BOYLAND, E.—(1935) *J. Path. Bact.*, **41**, 231.
CHIECO-BIANCHI, L., DE BENEDICTIS, G., TRIDENTE, G. AND FIORE-DONATI, L.—(1963) *Br. J. Cancer*, **17**, 672.
DE BENEDICTIS, G., MAIORANO, G., CHIECO-BIANCHI, L. AND FIORE-DONATI, L.—(1962) *Br. J. Cancer*, **16**, 686.
DELLA PORTA, G., CAPITANO, J., PARMI, L. AND COLNAGHI, M. I.—(1967) *Tumori*, **53**, 81.
FIORE-DONATI, L., CHIECO-BIANCHI, L., DE BENEDICTIS, G. AND MAIORANO, G.—(1961) *Nature, Lond.*, **190**, 278.
HOWELL, J. S.—(1961) *Br. J. Cancer*, **15**, 263.
KAYE, A. M.—(1960) *Cancer Res.*, **20**, 237.

Liebelt, R. A., Yoshida, R. and Gray, G. F.—(1961) *Proc. Am. Ass. Cancer Res.*, **3**, 245.
Mori, K.—(1963) *Gann*, **54**, 415.
Nettleship, A. and Henshaw, P. S.—(1943) *J. natn. Cancer Inst.*, **4**, 309.
Pietra, G., Rappaport, H. and Shubik, P.—(1961) *Cancer, N.Y.*, **14**, 308.
Saffiotti, U., Montesano, R., Sellakumar, A. R. and Borg, S. A.—(1967) *Cancer, N.Y.*, **20**, 857.
Shabad, L. M.—(1962) *J. natn. Cancer Inst.*, **28**, 1305.
Takayama, S. and Oota, K.—(1965) *Gann*, **56**, 189.
Tannenbaum, A. and Silverstone, H.—(1958) *Cancer Res.*, **18**, 1225.
Thornton, T. F. Jr. and Adams, W. E.—(1944) *Cancer Res.*, **4**, 55.
Trainin, N., Precerutti, A. and Law, L. W.—(1964) *Nature, Lond.*, **202**, 305.